# The Numerous Benefits of Social Media for Medicine. Comment on “Documenting Social Media Engagement as Scholarship: A New Model for Assessing Academic Accomplishment for the Health Professions”

**DOI:** 10.2196/27664

**Published:** 2021-06-09

**Authors:** John Alan Gambril, Carter J Boyd, Jamal Egbaria

**Affiliations:** 1 Department of Internal Medicine Ohio State University Wexner Medical Center Columbus, OH United States; 2 Nationwide Children's Hospital Columbus, OH United States; 3 Hansjörg Wyss Department of Plastic Surgery NYU Langone Health New York, NY United States; 4 University of Alabama at Birmingham School of Medicine Birmingham, AL United States

**Keywords:** social media, medical education, internet, academic medicine, promotion, tenure, health professions, scholarship, medicine, research, accomplishment, crowd source, contribution, innovation, education, dissemination

In their recent paper, Acquaviva et al [1] developed a set of guidelines to standardize curriculum vitae (CV) documentation of scholarly contributions made via social media platforms. Appropriately, the authors crowd-sourced contributions for the guidelines from the popular social media platform Twitter. Their work underscores the value of social media in idea sharing, highlights the growing role of online platforms in medical education, and signifies an important step in modernizing academic recognition to match the modernization of current medical learners.

Social media offers numerous scholarly and professional benefits [2,3]. These platforms have grown popular among the academic and medical communities as they are a means of networking with colleagues around the globe, discussing hot topics in various fields, engaging in medical education, sharing experiences through narrative medicine, and disseminating information to the lay-public [4]. More recently, social media has also taken on a vital role in residency recruitment. Without in-person interviews, programs have had to adopt new methods of sharing program strengths, highlighting unique program qualities, and appealing to applicants at an individual level [5].

We are avid proponents of academic social media. We can anecdotally attest to the educational value that arises from academic posts and discourse on social media platforms. Twitter, for example, offers a highly diverse pool of opinions covering all niches of medicine. It allows communication between individuals who might otherwise never interact. Sharing articles of interest via “tweeting” brings primary literature to your network’s fingertips. Twitter brings full professors and first-year medical students into the same arena of idea sharing. In a world that has increasingly recognized the shortcomings of traditional didactic lectures, social media offers modern educational methods better suited for today’s learners. Examples of this include “Tweetorials” (educational threads exploring a particular topic or phenomenon), podcasts, infographics, blogs, and virtual journal clubs among others [4].

These innovative methods of education through social media are intriguing to passionate educators. Those who seek to share knowledge and contribute to the advancement of scholarship will teach in whatever methods are most effective and will reach the most pupils. Importantly, social media is free, offering accessible medical education in a climate rife with expensive online materials and rising tuition. The value of these academic contributions must not go unrecognized. The time and dedication that goes into the development of educational posts through innovative methods should not be left out of an individuals’ portfolio simply because the medium is not classic. If anything, the ingenuity and adaptability of the medium creates added value to the material. As the face of education evolves with our digital world, propelled forward by the COVID-19 pandemic, academia must evolve in unison to recognize these contributions.

We are thankful to Acquaviva and associates [1] for providing us with much-needed guidelines that allow for the documentation of education portfolios representative of today’s evolving medical education environment.

## Abbreviations

CV: curriculum vitae
